# Autoimmune Disease Classification by Inverse Association with SNP Alleles

**DOI:** 10.1371/journal.pgen.1000792

**Published:** 2009-12-24

**Authors:** Marina Sirota, Marc A. Schaub, Serafim Batzoglou, William H. Robinson, Atul J. Butte

**Affiliations:** 1 Stanford Center for Biomedical Informatics Research, Stanford University School of Medicine, Stanford, California, United States of America; 2 Department of Pediatrics, Stanford University School of Medicine, Stanford, California, United States of America; 3 Lucile Packard Children’s Hospital, Palo Alto, California, United States of America; 4 Computer Science Department, Stanford University, Stanford, California, United States of America; 5 Division of Immunology and Rheumatology, Stanford University School of Medicine, Stanford, California, United States of America; 6 Geriatric Research Education and Clinical Center, Veterans Affairs Palo Alto Health Care System, Palo Alto, California, United States of America; University of Alabama at Birmingham, United States of America

## Abstract

With multiple genome-wide association studies (GWAS) performed across autoimmune diseases, there is a great opportunity to study the homogeneity of genetic architectures across autoimmune disease. Previous approaches have been limited in the scope of their analysis and have failed to properly incorporate the direction of allele-specific disease associations for SNPs. In this work, we refine the notion of a genetic variation profile for a given disease to capture strength of association with multiple SNPs in an allele-specific fashion. We apply this method to compare genetic variation profiles of six autoimmune diseases: multiple sclerosis (MS), ankylosing spondylitis (AS), autoimmune thyroid disease (ATD), rheumatoid arthritis (RA), Crohn's disease (CD), and type 1 diabetes (T1D), as well as five non-autoimmune diseases. We quantify pair-wise relationships between these diseases and find two broad clusters of autoimmune disease where SNPs that make an individual susceptible to one class of autoimmune disease also protect from diseases in the other autoimmune class. We find that RA and AS form one such class, and MS and ATD another. We identify specific SNPs and genes with opposite risk profiles for these two classes. We furthermore explore individual SNPs that play an important role in defining similarities and differences between disease pairs. We present a novel, systematic, cross-platform approach to identify allele-specific relationships between disease pairs based on genetic variation as well as the individual SNPs which drive the relationships. While recognizing similarities between diseases might lead to identifying novel treatment options, detecting differences between diseases previously thought to be similar may point to key novel disease-specific genes and pathways.

## Author Summary

In this work, we define a novel concept of a disease variation profile and carry out comparative analyses to find similarities and differences in the genetic architectures of common diseases. Studying genetic variation across autoimmune disease in particular allows us to systematically identify allele-specific relationships. We find polymorphisms where the alleles are significantly similarly associated with multiple autoimmune conditions as well as polymorphisms where alleles are significantly differentially associated with multiple conditions. We apply this method to compare genetic variation profiles of six autoimmune diseases: multiple sclerosis (MS), ankylosing spondylitis (AS), autoimmune thyroid disease (ATD), rheumatoid arthritis (RA), Crohn's disease (CD), and type 1 diabetes (T1D), as well as five non-autoimmune diseases. We find two autoimmune disease groups where SNPs that make an individual susceptible to one class of autoimmune disease also protect from diseases in the other autoimmune class. As more genome-wide association data becomes available, future studies could be done across tens or hundreds of diseases yielding the commonalities and differences in genetic architectures across all of human disease.

## Introduction

Autoimmune diseases share many genetic factors resulting in similarity of disease mechanisms. For instance the HLA region is known to be associated with several autoimmune diseases including T1D, MS, RA as well as others [1],[2]. Certain autoimmune diseases, such as MS and ATD [3], T1D and celiac disease [4] commonly co-occur in patients [5],[6]. Classes of drugs, for instance steroids, are known to treat groups of inflammatory and autoimmune conditions such as RA, CD, MS and systemic lupus erythematosus.

Despite these similarities, there is evidence that points towards genetic differences between autoimmune diseases. For instance *rs2076530* (A/G), a single nucleotide polymorphism (SNP) in *BTNL2* (butyrophilin-like 2, a MHC class II associated gene), has been shown to be strongly associated with several autoimmune diseases such as MS, RA, T1D, sarcoidosis and systemic lupus erythematosus (SLE) [7]–[12]. A more in depth analysis shows that while the G allele of the polymorphism was more frequent among patients with T1D and RA than healthy controls, the A allele was more frequent in patients with SLE than in healthy individuals [12]. This example demonstrates the idea that while a single SNP might be significantly associated with several disorders, an allele could make an individual susceptible to one disease, but be protective of another. Finally we know that despite the common mechanisms of autoimmune diseases, there are drugs that treat one autoimmune condition, but unexpectedly worsen another. For instance infliximab, an anti-TNF agent, has been demonstrated to offer benefits for the treatment of some autoimmune disorders, such as RA and AS [13],[14], but it fails or even exacerbates the condition in patients with other disorders such as MS [15]. Similarly interferon-beta, which is widely used to treat MS, has no effect on RA patients [16].

With multiple genome-wide association studies (GWAS) performed across autoimmune diseases, we have an ideal setup to study the homogeneity of genetic architectures across autoimmune disease. By sampling specific locations in the genome, the technology behind GWAS allows us to quickly and accurately analyze samples for genetic variations that contribute to disease predisposition. Since being introduced in 2007, GWAS have helped identify several hundred common marker alleles that are associated with over seventy different conditions [17]. Integrative meta-analyses have been carried out to analyze several GWAS to study a single disease of interest such as type II diabetes [18]. Genome-wide association (GWA) data has also been integrated with gene expression data to prioritize genes for disease association [19].

In this work, we define a novel concept of a disease variation profile and carry out comparative analyses to find similarities and differences in the genetic architectures of common diseases. Studying genetic variation in autoimmune diseases in particular allows us to systematically define a disease classification based on allele-specific relationships. We find individual polymorphisms where the same alleles are significantly associated with multiple autoimmune conditions as well as polymorphisms where different alleles are significantly associated with multiple conditions.

Several measures of association are commonly used to quantify the relationship between a SNP and a disease phenotype. A p-value measures how much evidence there is against the hypothesis that the allele distribution in the control and disease populations is the same. An odds-ratio is the ratio of the probability that a disease individual has a certain allele to the probability of a healthy control having that allele. An odds-ratio of 1 implies that the allele is equally likely in both groups. An odds-ratio greater than one implies that the allele is more likely in the disease group. Similarly, an odds-ratio less than one implies that the allele is less likely in the disease group. While the odds-ratio doesn’t reflect the sample size of the study, the width of the confidence interval on the odds-ratio is reflective of sample size. The odds-ratio furthermore allows us to specify which allele is associated with the disease and how strong that association is.

Recent studies explored the genetic relationships between seven common diseases studied by Wellcome Trust Case Control Consortium (WTCCC) [20] based on similarities of associated genes and their pathways [21]–[23]. Previous approaches use p-values to measure the significance of the association between a SNP and a single disease from genome-wide association data, and compute a measure of similarity between these p-values in pairs of diseases. While these approaches are able to identify pairs of diseases that have similar genetic variation profiles based purely on strength of association of each loci, the metric is not allele-specific, meaning it does not distinguish between which of the two alleles is associated with a disease. In our own previous work, we have used a classifier approach in order to discover similarities in disease variation profiles [24] by examining a large number of SNPs for each individual instead of analyzing the significance of individual SNPs across diseases. While successful in finding similarities between diseases, the classifier approach requires individual genotype data to be carried out on the same platform.

In this paper, we present a novel, allele-specific, cross-platform method for comparing genetic architecture of disease for which GWA data is available. Our approach relies on the raw summary statistics of genome-wide association studies and does not require obtaining individual level genotype data. As a result, our approach allows for data to be combined across different platforms. We define a genetic variation score (***GVS***) for each SNP-disease pair as a combination of the p-value to represent the strength of association between the SNP and the disease phenotype and the odds-ratio to specify which allele is the one associated with the disease (see Methods). We define a genetic variation profile for a disease as a vector of the ***GVS*** values across all the measured SNPs. We use the genetic variation profiles to discover allele-specific relationships between disease pairs.

We apply our method to a combined dataset of two WTCCC [20],[25] studies to uncover positive and negative disease relationships within six autoimmune diseases, multiple sclerosis (MS), ankylosing spondylitis (AS), autoimmune thyroid disease (ATD), rheumatoid arthritis (RA), Crohn's disease (CD), and type 1 diabetes (T1D), and five non-autoimmune diseases, bipolar disorder (BD), coronary artery disease (CAD), hypertension (HT), type 2 diabetes (T2D), and breast cancer (BC). Applying our method to this broad panel, we expected to find all the known autoimmune diseases clustered similarly. However, we find two separate classes of autoimmune disease. RA and AS fall into one class, while MS and ATD into the other. T1D is similar to ATD, but not MS and therefore is difficult to classify. CD is similar to none of the other five autoimmune diseases and thus is not further discussed with the other autoimmune diseases. We identify specific SNPs and genes with similar and opposite risk profiles for these two classes of autoimmune disease and suggest differing mechanisms of disease and strategies for future drug development for the two classes.

## Results

In this work, we analyze genome-wide association data across a set of eleven conditions to find allele-specific similarities and differences across disease. Our combined dataset includes six autoimmune diseases (MS, AS, ATD, RA, CA and T1D) and five non autoimmune diseases (BC, BD, CAD, HT and T2D). We added independent GWA studies for two autoimmune diseases: RA from North American Rheumatoid Arthritis Consortium (NARAC) and the Swedish Epidemiological Investigation of Rheumatoid Arthritis (EIRA) [26] and MS from the International Multiple Sclerosis Genetics Consortium (IMSGC) [27]. In order to be able to compare genetic variation profiles across eleven diseases on different platforms, we only consider 573 SNPs that are commonly measured in these datasets (see Methods). The distribution of these SNPs throughout the genome (Figure S1) does not exhibit a visible bias. We furthermore carry out several experiments in order to assess the validity of using a small subset of SNPs to obtain our findings (see Discussion).

By examining the strength of association of each SNP with each disease (p-value), we found a set of SNPs which are significantly associated with all 5 autoimmune diseases in our dataset (Table 1). When we examined the odds-ratios for these SNPs, we saw that individual alleles are oppositely associated with different autoimmune diseases. Our analysis supports the fact that simple consideration of p-values as a genetic variation profile of a disease is not sufficiently representative of the potential disease mechanisms.

**Table 1:** SNPs significantly associated with RA, AS, T1D, MS, and ATD (p<0.05).

SNP - Allele	Gene Symbol	Sign of the Odds-Ratio | P-value									
		RA		AS		T1D		ATD		MS	
**rs1063635 – A**	LOC100129668	−	6.01E-08	+	1.83E-59	−	8.65E-10	−	8.30E-03	−	9.27E-05
**rs1132200 – A**	TMEM39A	−	2.24E-02	−	1.77E-02	−	8.28E-03	−	4.02E-03	−	4.56E-03
**rs1634717 – A**		−	1.80E-04	+	6.00E-13	+	4.94E-14	+	1.68E-06	+	3.34E-03
**rs204991 – C**	GPSM3	−	3.67E-08	−	9.34E-13	+	9.40E-24	+	5.04E-11	−	1.70E-03
**rs2076530 – G**	BTNL2	+	3.50E-57	+	8.76E-15	+	2.64E-14	−	3.93E-07	−	3.00E-19
**rs2242655 – C**	C6orf47	+	1.21E-03	+	5.75E-23	+	1.53E-05	−	1.13E-02	−	7.42E-05
**rs2248462 – A**		−	1.33E-03	+	1.09E-99	−	5.00E-25	−	1.45E-05	−	5.94E-19
**rs2299851 – T**	MSH5	+	4.91E-02	+	1.10E-22	+	1.04E-04	−	4.69E-02	−	5.52E-06
**rs2517646 – G**	TRIM10	−	1.98E-04	−	2.29E-03	−	1.86E-06	−	1.26E-02	+	7.59E-06
**rs2844463 – T**	BAT3	−	6.40E-07	−	1.56E-04	+	1.47E-05	−	1.15E-02	+	3.70E-02
**rs3129953 – T**	BTNL2	−	2.54E-11	−	2.13E-09	+	1.47E-40	+	2.66E-15	−	4.18E-05
**rs3135363 – C**		−	5.69E-22	−	7.21E-04	+	9.81E-12	+	4.46E-15	−	5.11E-07
**rs4428528 – C**		−	1.01E-18	−	2.19E-03	+	8.16E-23	+	7.11E-12	−	1.22E-03
**rs887464 – A**	PSORS1C3	+	3.20E-03	+	7.89E-09	+	7.43E-28	+	2.03E-05	−	7.28E-10
**rs9267954 – T**		+	2.89E-38	+	3.27E-13	+	4.40E-12	−	2.50E-02	−	2.17E-14

SNPs that are significantly associated with all five autoimmune diseases (based on p-values previously reported by the WTCCC). While these SNPs are commonly significantly associated with five autoimmune diseases in our dataset, by examining the signs of the odds-ratios we see that for the same SNP, often different alleles are associated with different diseases.

For each disease-SNP pair, we define a new genetic variation score (***GVS***) by combining both the uncorrected p-value to capture the strength of association, and the odds-ratio, to account for which allele is associated with a given disease (see Methods). For each disease, a genetic variation profile consists of ***GVS*** for all the SNPs commonly measured across our input GWAS. We use Pearson correlation to measure allele-specific similarities and differences between disease pairs. In order to test the significance of our findings, we compute the false discovery rate (FDR) for the correlations by comparing the actual distribution of correlations to that calculated on a randomized dataset (see Methods). To identify relationships between groups of diseases, hierarchical cluster analysis is applied to the data using the computed Pearson correlation coefficients as a distance metric between disease pairs. In order to confirm our findings, we included additional independent studies of RA and MS in our analysis [26],[27].

**Figure 1:**
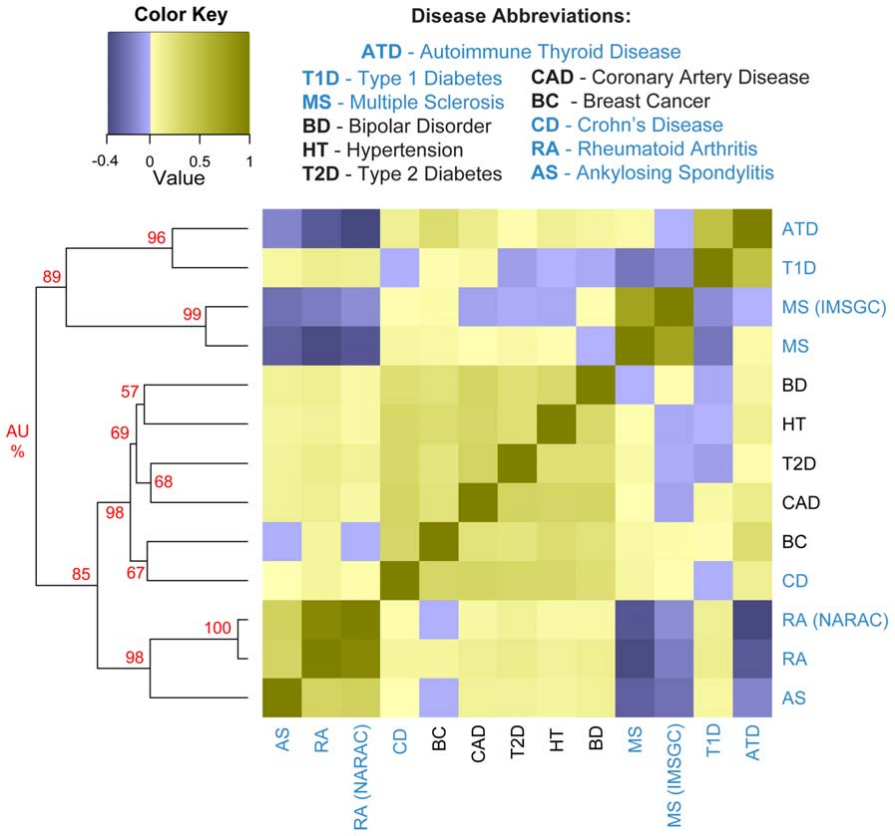
Disease heatmap based on genetic variation profiles. This diagram shows correlations between disease genetic variation profiles. Positive relationships between a pair of diseases are shown in brown, negative relationships are shown in purple. The diseases highlighted in blue have an autoimmune component. Hierarchical clustering using these correlations as a distance metric is shown on the left. Approximately Unbiased (AU) probability values (%) for each cluster indicating how strongly the cluster is supported by data are shown in red. Clusters with AU larger than 95% are strongly supported by data.

The comparison between genetic variation profiles of 11 diseases is shown in Figure 1 (Table S1) with the corresponding FDR (Table S2). We can see that there are two main groupings of autoimmune diseases, with T1D showing similarity to both groupings, and CD to neither. Although CD is an inflammatory disease affecting the gastrointestinal tract with an autoimmune component, as we did not see a strong relationship between CD and the other set of autoimmune diseases in our analysis, we did not consider it with the rest of the autoimmune diseases in our further discussion. We also notice that the non-autoimmune diseases are clustered together. HT, BD, T2D, CAD and BC are all slightly positively correlated. One implication of the positive correlations between these diseases is that there might be a common underlying genetic variation profile of disease. While we are interested in exploring this notion in the future, we focus our discussion here on the stronger and more surprising relationships between autoimmune diseases that we find.

Both RA datasets and AS have similar genetic variation profiles (Pearson correlation 0.340 and 0.357) and are negatively correlated with genetic variation profiles of both MS datasets and ATD (Pearson correlation −0.42 and −0.353). Out of 573 SNPs that are commonly measured in all the datasets, we find a set of nine SNPs such that one allele predisposes an individual to one class of autoimmune diseases, but protects from the other class (Table 2). When a similar analysis was carried out on randomized null data, over 100 trials, on average less than a single SNP is found using the same criteria. While this relationship has been previously established for *rs2076530* in *BTNL2* in a subset of the autoimmune diseases [12], we systematically identify all such SNPs which are significantly associated with at least one disease per class (Table 2). Some of these regions have previously been associated with autoimmunity; for example *rs10484565* falls in a gene called *TAP2*, which encodes a membrane-associated protein that is a member superfamily of ATP-binding cassette (ABC) transporters. While mutations in this gene have been previously associated with ankylosing spondylitis, insulin-dependent diabetes mellitus, and Grave’s Disease [28]–[30], the inverse allelic relationship has not been previously recognized. *rs1265048* falls near *CDSN* and *PSORS1C1* both of which have previously been associated with susceptibility to psoriasis [31],[32]. *rs151719* falls in *HLA-DMB*, an MHC class II molecule that has been previously associated with T1D [33]. We hypothesize that there are loci which pre-dispose individuals to autoimmune disease in general (such as *rs1132200* in *TMEM39A* in Table 1) and other loci that determine which class or more specifically which autoimmune disease an individual is more likely to get (Table 2).

**Table 2:** SNPs with opposite risk profiles in two autoimmune classes.

SNP - Allele	Gene Symbol	Genetic Variation Score (GVS)						
		RA (NARAC)	RA	AS	T1D	ATD	MS (IMSGC)	MS
**rs11752919 - C**	ZSCAN23	−3.48	−3.21	−9.39	1.10	0.70	3.25	2.99
**rs3130981 - A**	CDSN	−0.46	−1.00	−9.47	−4.94	0.33	10.00	13.41
**rs151719 - G**	HLA-DMB	−6.71	−4.77	−1.08	−13.63	0.34	8.58	17.76
**rs10484565 - T**	TAP2	25.52	8.37	1.34	15.74	−1.36	−0.56	−0.30
**rs1264303 - G**	VARS2	11.51	7.36	18.76	0.89	−1.76	−1.85	−1.75
**rs1265048 - C**	CDSN	6.59	2.97	50.13	6.34	−0.85	−2.39	−4.16
**rs2071286 - A**	NOTCH4	5.30	0.78	6.42	4.04	−0.03	−1.89	−2.45
**rs2076530 - G**	BTNL2	67.49	56.46	14.06	13.58	−6.41	−9.50	−18.52
**rs757262 - T**	TRIM40	14.58	9.11	6.27	1.56	−0.79	−2.05	−7.34

SNPs such that one allele predisposes an individual to one class of autoimmune diseases (RA and AS), but protects from the other class (MS and ATD) or vice versa. Each SNP in this set has a significant association (p<0.05) with at least one disease per class. The SNPs where the minor allele has a negative odds-ratio (protective) are underlined to show the separation more clearly.

In discovering these two classes of autoimmune disease, we find positive and negative pair-wise relationships between genetic variation profiles of diseases. In this paper, we present the top ten disease pairings that are significantly correlated (FDR<0.01) from our dataset: NARAC RA and ATD (Pearson correlation −0.433), WTCCC RA and ATD (Pearson correlation −0.353), NARAC RA and WTCCC MS (Pearson correlation −0.367), WTCCC RA and WTCCC MS (Pearson correlation −0.42), AS and WTCCC MS (Pearson correlation −0.322), AS and IMSGC MS (Pearson correlation −0.256), WTCCC MS and T1D (Pearson correlation −0.229), T1D and ATD (Pearson correlation 0.49), WTCCC RA and NARAC RA (Pearson correlation 0.935), and WTCCC MS and IMSGC MS (Pearson correlation 0.717) (Table S1, highlighted in red).

### Negative Disease–Disease Relationships

A negative correlation between two genetic variation profiles means that while the two phenotypes have strong association with the same SNPs, alleles are oppositely responsible for predisposing an individual to each of the diseases. Therefore if two phenotypes have negatively correlated genetic variation profiles, some alleles that are susceptible to one phenotype are protective of the other and vice versa.

**Figure 2:**
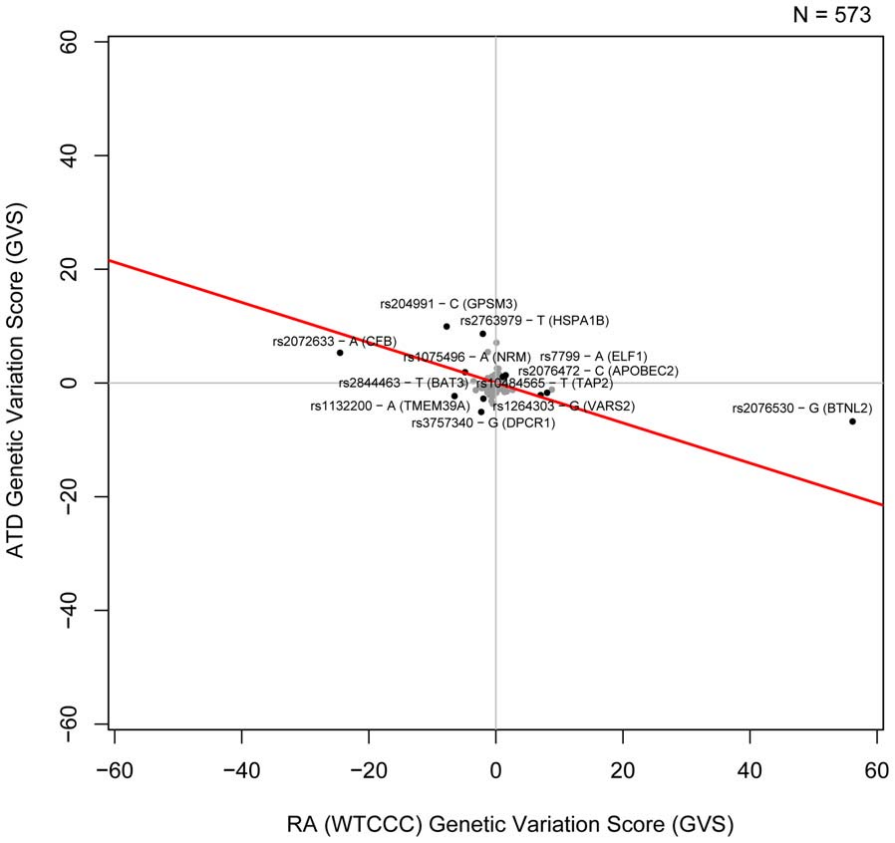
Genetic Variation Scores for RA (WTCCC) and ATD. Genetic Variation Scores (GVS) for SNPs that are significantly associated with both diseases (p<0.05) are shown in black. The non-significant GVS are shown in gray. The best fit linear model of the data is shown in red.

The strongest negatively correlated disease pair is ATD and the NARAC RA study with a correlation score of −0.433. This finding is supported by a strong negative correlation between ATD and the WTCCC RA genetic variation profiles (Pearson correlation −0.353). The average lowest negative correlation on randomized data from 100 trials was −0.13 with a standard deviation 0.09. RA is a chronic, systemic autoimmune disorder in which the immune system attacks the joints, causing joint inflammation and destruction. ATD, also referred to as Grave’s Disease, is caused by an antibody-mediated autoimmune reaction resulting in neck swelling, bulging eyes and hyperthyroidism. There is a known association between rheumatologic and thyroid disorders [34]. Early studies of autoimmune thyroid disease and thyroid auto-antibodies in rheumatoid arthritis patients suggest that there may be a common genetic link between RA and autoimmune thyroid disease [35],[36]. More recently it has been suggested that the abnormalities of the joints and thyroid gland are related most probably due to a genetic predisposition determined by the affiliation to a certain HLA type, most often *HLA-DR* [37]–[39]. We find a set of SNPs which are strongly associated with both ATD and RA but when we look at the allele-specific genotype counts for these loci, we see that while one of the alleles is more common in RA patients, the other is more common in ATD patients. The negative association trend between WTCCC RA and ATD and the individual SNPs contributing to the correlation are shown in Figure 2. Those include polymorphisms in complement factor B (*CFB*), nuclear envelope membrane protein (*NRM*), heat shock protein (*HSPA1B*) as well as others. Similarly, the significant negative association trend between NARAC RA and ATD is shown in Figure S2.

**Figure 3:**
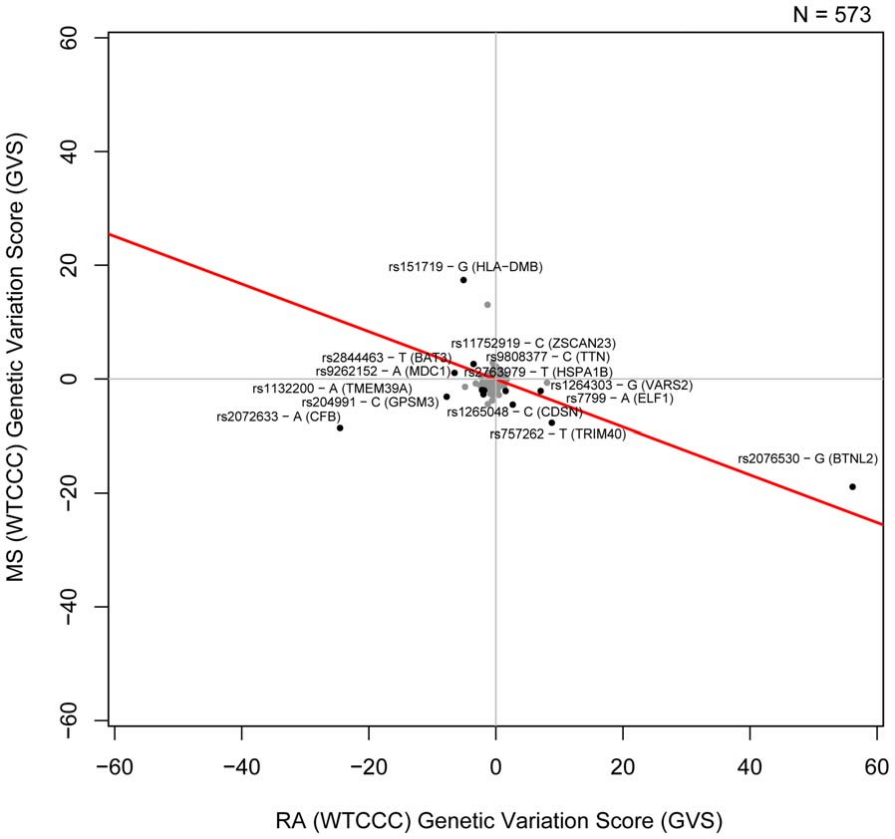
Genetic Variation Scores for RA (WTCCC) and MS (WTCCC). Genetic Variation Scores (GVS) for SNPs that are significantly associated with both diseases (p<0.05) are shown in black. The non-significant GVS are shown in gray. The best fit linear model of the data is shown in red.

Multiple sclerosis (WTCCC) and rheumatoid arthritis (WTCCC) are significantly negatively correlated (Pearson correlation −0.42, Figure 3). This finding is supported by a significant negative correlation between NARAC RA and the WTCCC MS genetic variation profiles (Pearson correlation −0.367, Figure S3) as well as a weaker negative correlations between WTCCC RA and IMGSC MS as well as NARAC RA and IMSGC MS genetic variation profiles (Pearson correlations −0.204 and −0.141 respectively). Multiple sclerosis is an autoimmune condition in which the immune system attacks the myelin sheaths of the central nervous system. We have not been able to find any recorded co-occurrence of the two disorders from previous research. With the exception of the HLA region there has been very little work linking genetic susceptibility of these two immunological disorders. We identify a set of SNPs for which an allele predisposes an individual to RA while being protective of MS and vice versa. The negative association trend between RA and MS and the individual SNPs contributing to the correlation are shown in Figure 3. Those include polymorphisms in HLA-B associated transcript 3 (*BAT3*), E74-like factor 1 (*ELF1*), *HLA-DMB, VARS2, BTNL2, TRIM40, ZSCAN23* and *CDSN*.

**Figure 4:**
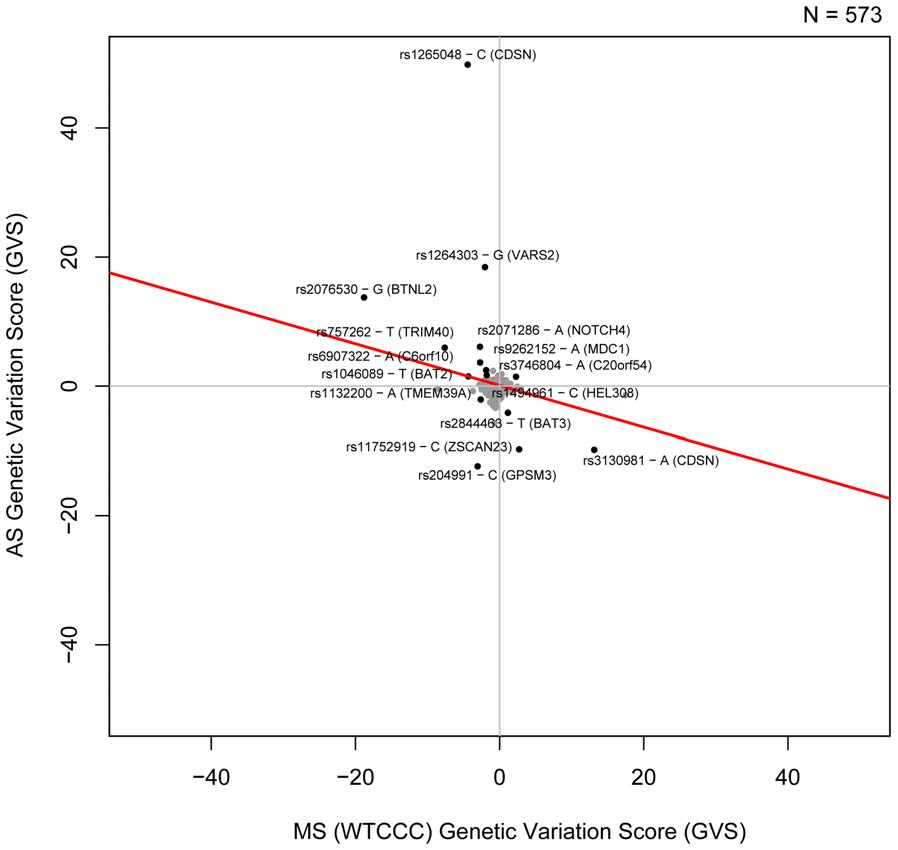
Genetic Variation Scores for AS and MS (WTCCC). Genetic Variation Scores (GVS) for SNPs that are significantly associated with both diseases (p<0.05) are shown in black. The non-significant GVS are shown in gray. The best fit linear model of the data is shown in red.

Similarly, the genetic variation profiles of AS and WTCCC MS are negatively correlated (Pearson correlation −0.322, Figure 4). This finding is supported by a significant negative correlation between AS and the IMSGC MS genetic variation profiles (Pearson Correlation -0.256, Figure S4). Ankylosing spondylitis is a systemic rheumatic disease resulting in chronic inflammation of the spine and the sacroiliac joints. Several individual loci have been linked to both disorders but overall association has not been previously established. For instance, while association of the *IL23R* gene with inflammatory bowel disease, psoriasis and ankylosing spondylitis [25] has been shown before, only recently has its involvement also been linked to MS [40]. We identify a set of SNPs for which one allele predisposes an individual to AS while being protective of MS, and vice versa. These include polymorphisms in mediator of DNA-damage checkpoint 1 (*MDC1*), HLA-B associated transcript 2 (*BAT2*), as well as others.

### Positive Disease–Disease Relationships

A positive correlation between two genetic variation profiles means that not only the same SNPs, but also the same alleles lead an individual to be more susceptible to both phenotypes.

**Figure 5:**
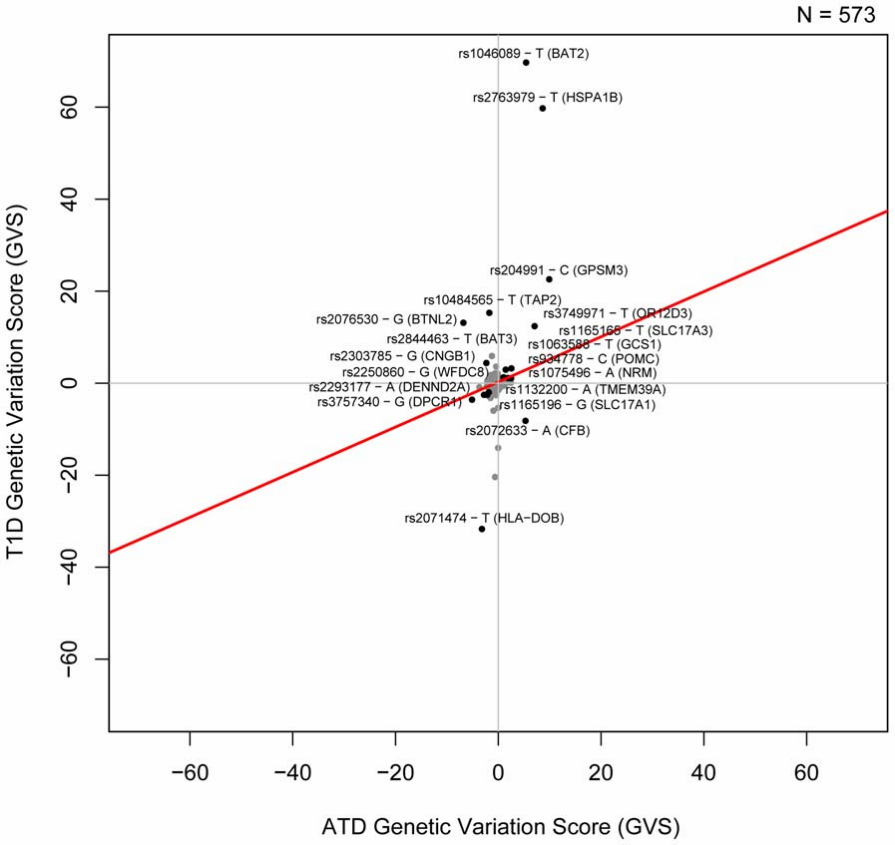
Genetic Variation Scores for ATD and T1D. Genetic Variation Scores (GVS) for SNPs that are significantly associated with both diseases (p<0.05) are shown in black. The non-significant GVS are shown in gray. The best fit linear model of the data is shown in red.

The highest non-obvious positive correlation of 0.481 is between T1D and ATD. The average highest positive correlation on randomized data from 100 trials was 0.12 with a standard deviation 0.08. The positive association trend as well as individual data points can be seen on Figure 5. T1D is an autoimmune disease that results in destruction of insulin-producing beta cells of the pancreas. Several recent studies reported shared variants among these autoimmune disorders [41],[42]. There is increasing evidence that autoimmune thyroid disease is frequent in patients with T1D [43],[44]. Co-occurrence of T1D and ATD in the same patient or family has also been studied from the epidemiological perspective resulting in finding several common susceptibility genes [45]. Two loci that have previously been reported to be associated with T1D were recently shown to also be significant risk factors for the co-occurrence of ATD and T1D in Japanese individuals [46]. We identify over a dozen other loci, mostly in the HLA region, which are commonly associated between the two diseases.

The overall strongest positive correlations, as expected, are those between the two RA (WTCCC and NARAC) and the two MS (WTCCC and IMSGC) datasets (Pearson correlation coefficients of 0.935 and 0.717 respectively), which confirms our hypothesis. Figures S5 and Figure S6 show the strong positive correlation between the genetic variation profiles of the two RA and MS studies respectively. This result supports the proposed design for a disease-specific genetic variation profile and the comparison metric used in the analysis.

## Discussion

In this work, we present a novel notion of a genetic variation profile and apply it to carry out comparative analysis of a set of eleven diseases. Half of these diseases are known to have an autoimmune component including RA, T1D, AS, MS and ATD. Our analysis yields several significant positive and negative relationships between these diseases. We identify two broader classes of autoimmune disease (RA and AS fall into one, and MS and ATD into the other) as well as a set of SNPs which when predisposing an individual to one class of the diseases protects from the second. We explore individual SNPs and genes that play an important role in defining similarities and differences between disease pairs.

We find that p-values, as a measure of association, fail to capture which allele is susceptible and which is protective and are thus not a good metric for studying similarities and differences in disease genetic variation profiles. We introduce a novel notion of a genetic variation score **(** ***GVS*** **)** which captures both the strength of association of a given SNP and whether an allele is protective or susceptible. Using this measure we are able to identify new positive and negative relationships between disease pairs as well as identify individual SNPs which drive the relationships such as previously reported *rs2076530* in *BTNL2*, in which the G allele predisposes to RA, AS and T1D, but protects from MS and ATD [12].

We have shown that studying genetic variation across autoimmune diseases in particular allows us to systematically identify allele-specific pleiotropic effects. We find that the same allele can be associated with multiple phenotypes. A likely explanation for the same SNP allele being associated with different phenotypes is that it interacts differentially with genetic and environmental factors that change the biological context of the SNP in different individuals. More importantly, we find that certain alleles can be disease-associated in one setting and disease-protective in another. We hypothesize that there are some loci which pre-dispose individuals to disease in general, and other loci that determine which class or more specifically which disease an individual is more likely to get.

More specifically, we find that certain MHC polymorphisms predispose individuals to one class of autoimmune disease but are protective against the other (Table 2). We hypothesize that this could be due to their involvement in peptide-MHC loading. For instance *HLA-DM* (*rs151719*), a chaperone binder for nascent MHC molecules, could differentially modulate peptide binding and thus antigen presentation. *TAP2* (*rs10484565*) is also involved in transporting peptides from the cytoplasm to the ER to couple them with nascent MHC molecules. Both *HLA-DM* and *TAP2* are involved in peptide-MHC loading, which could explain their diametric effects. These MHC chaperone binders might load pathogenic peptides for one disease but not another. *CDSN* (*rs1265048*) is also located in the MHC on chromosome 6, but has not been described to alter antigen presentation.

Phenotypic expression of variant alleles is influenced differentially by environment, stochastic events, and interactions with multiple other genetic loci. Traditional SNP analysis does not account for gene interactions, however gene interactions are instrumental for understanding principles for how, when and why genetic variation is phenotypically expressed [47]. We show in this report that genetic variants are expressed differentially, with respect to human disease, presumably due to the combined action of different alleles of several genes. However, the molecular basis of such gene interactions remains only speculative [48]. Phenotypic expression depends on the environmental and genetic context of a biological system. Borrowing from the literature in microbial systems biology, these can be viewed as constraints on the biological system in question [49]. The environmental and genetic constraints of one disease may be highly inconsistent with another. Though they share a common SNP, the particular allele may act as an ‘on switch' or alternatively an 'off' switch in making an individual more or less susceptible to disease.

Classification of diseases based on allelic differences may be used in the future to illuminate potential new therapies. Certain drugs like anti-TNF have positive effects in RA, psoriasis and ankylosing spondylitis as compared to MS suggesting that certain molecules may have diametric roles in different diseases [50]. Assuming specific alleles of genes that are useful for classifying diseases reflect an underlying biological process, then it follows that drugs useful for treating any particular disease may be useful for treating another disease in its class. Thus, integration of clinical correlates with genomic sub-classification of diseases could be a useful and relatively straightforward strategy for personalized medicine.

There are a few limitations to our current approach that should be recognized. The data for the analysis is obtained from several types of arrays. While our approach allows for data analysis across multiple platforms, it is dependent on the intersection of coverage between all those platforms. The overlap coverage in terms of SNPs between all platforms that we currently analyze is minimal (573 SNPs). A larger overlap could be obtained by using linkage disequilibrium and taking advantage of SNPs in the same haplotype blocks or by applying imputation techniques, but we chose to rely solely on the data available to us, until confidence in imputation methods improves. In addition while our current approach relies solely on the summary statistics data, introducing an imputation step in our pipeline would require us to obtain and incorporate individual genotype data, which we see as a drawback.

In order to assess the validity of using a small subset of SNPs to obtain our findings, we repeated the experiment considering only the diseases (RA, HT, T1D, T2D, CAD, CD and BD) for which the genotyping was done using Affymetrix GeneChip 500K Mapping Array Set across nearly 500,000 measured SNPs. This allowed us to compute similarities between genetic variation profiles using all the SNPs on the array. We find that the pair-wise correlations resulting from this analysis are very similar to those obtained using only the 573 overlapping SNPs (Pearson correlation 0.88). This also holds for the diseases (MS, AS, ATD and BC) genotyped using the custom Illumina Infinium array across nearly 15,000 measured SNPs (Pearson correlation 0.98). Therefore we show that the pair-wise disease correlations that we compute using the common subset of 573 SNPs can be extrapolated to a genome-wide scale to draw conclusions regarding disease classification. The overlap problem will improve as more data on more common platforms becomes available in the future, and as more individuals are tested using whole genome sequencing. We also acknowledge that there are other more sophisticated statistical methods to compare genetic architectures and to cluster genetic variation profiles, however we picked a simple parsimonious approach to test our hypothesis.

In conclusion, we present a novel, systematic, cross-platform methodology to identify allele-specific relationships between disease pairs based on genetic variation as well as the individual SNPs which drive the relationships. We apply this method to compare genetic variation profiles of eleven diseases across several independent studies. We find two autoimmune disease groups where SNP alleles that make an individual susceptible to one class of autoimmune disease also protect from diseases in the other autoimmune class. Further integration of different types of biomedical data will improve our ability to conjure biological explanations for findings from GWAS. For instance, correlating genetic variation to gene expression might help interpret the molecular and genetic complexity of human disease [51]. As more GWA data becomes available, our method could be applied across tens or hundreds of diseases yielding the commonalities and differences in genetic architectures across all of human disease.

## Methods

The data for our analysis was obtained from two separate Wellcome Trust Case Control Consortium (WTCCC) studies: a GWA study of 2,000 cases and 3,000 shared controls for 7 complex human diseases (BD, CAD, CD, HT, RA, T1D and T2D) carried out with the Affymetrix GeneChip 500K Mapping Array Set, which comprises 500,568 SNPs and an association study of 1,500 common controls 1,000 cases for each of BC, ATD, AS, and MS carried out with a custom-made Illumina Infinium array with 14,436 non-synonymous SNPs. We introduce a second rheumatoid arthritis (1522 cases and 1850 controls) [26] as well as another multiple sclerosis (931 cases and 2431 controls) [27] GWA datasets to our analysis to further support our findings. The rheumatoid arthritis study combines data from North American Rheumatoid Arthritis Consortium (NARAC) and the Swedish Epidemiological Investigation of Rheumatoid Arthritis (EIRA) to genotype 317,503 SNPs using several versions of Illumina Infinium BeadChips. 297,086 SNPs that passed filters in both the NARAC and EIRA sample collections were merged into a single dataset for analysis. The MS study, carried out by the International Multiple Sclerosis Genetics Consortium (IMSGC) examined a set of 334,923 SNPs using Affymetrix GeneChip 500K Mapping Array Set.

We used the pre-computed p-values from the above experiments in our analysis. The smaller and larger WTCCC studies used the snpMatrix [52] and the PLINK [53] programs respectively to calculate a p-value for the strength of association between a SNP and a disease. For the independent RA analysis PLINK program [53] was used to carry out Cochran-Mantel-Haenszel stratified analysis and for the independent MS analysis transmission disequilibrium test was applied using the Whole-genome Association Study Pipeline (WASP) as well as PLINK [53]. The odds-ratios were re-computed using the genotype counts provided in the summary statistics for each experiment.

We start out by finding the SNPs common to both experiments. Each SNP is mapped to Entrez GeneID and corresponding gene symbol by querying dbSNP. SNPs that do not fall within a gene are not assigned one. The intersection of the two WTCCC studies as well as the two additional GWA datasets results in a set of 573 SNPs, which we use for our analysis. The distribution of these SNPs in the genome is shown in Figure S1. We confirm that each of the studies integrated in the pipeline have the data encoding on the same DNA strand by keeping track which allele was measured and comparing the counts of individuals with each measured allele for the control populations. For each SNP we consider the disease associations with respect to the minor allele. Please note that in some studies, the major allele is actually the risk allele therefore what we refer to as risk and protective may be different from the published studies of these phenotypes.

We define the notion of a genetic variation profile as a combination of log-odds scores and p-values for each SNP measuring allele-specific association between the SNP and each of the eleven diseases in the combined dataset. Using p-values alone as proposed previously by Torkamani et al. [21] does not capture which allele is associated with a given disease, as shown in Table 1. Log odds-ratios alone do not account for significance of association due to sample size. A typical relationship between log odds-ratios and log p-values are shown by a volcano plot (see Figure S7). The alleles for which the log odds-ratio is negative signify that the allele tested is less likely to appear in a disease individual (left half of the plot). The alleles for which the log odds-ratio is positive signify that the allele tested is more likely to appear in a disease individual (right half of the plot). From a volcano plot we can also see that p-values and odds-ratios are not necessarily correlated. One can envision a situation where a rare allele has a high magnitude odds-ratio with respect to a disease phenotype, but a poor p-value. These values fall into the lower corners of a volcano plot (Figure S7). Similarly, if the sample size is very large, a SNP that has a small effect might have a significant p-value, but an odds-ratio close to 1. Such values would fall into the top center of a volcano plot (Figure S7). A combination of p-values and odds-ratios is needed in order to capture the strength and direction of association between a SNP and a disease. Therefore we propose combining these two measures of association to represent a disease genetic variation profile. More specifically for each disease *d* and SNP *s*, we define a genetic variation score ***GVS[d,s]*** where *d* (number of diseases) = 1…n and *s* (number of SNPs) = 1…m: ***GVS[d,s]*** = sign(log(odds-ratio[d,s]))*(log(p-value[d,s])).

With respect to the minor allele, an odds-ratio greater than one implies that the minor allele is more likely in the disease group. An odds-ratio less than one implies that the minor allele is less likely in the disease group, which means that the major allele is more likely in the disease group. Therefore by looking at the sign of the log of the odds-ratio we can specify which allele is the one associated with a disease. We capture the significance and strength of that association by multiplying the sign of the log of the odds-ratio by the log of the p-value. After computing the ***GVS*** for each disease-SNP pair, we define a genetic variation profile for each disease as a vector of the ***GVS*** for all the measured SNPs. This allows us to capture strength of disease association across multiple SNPs in an allele-specific fashion.

As a similarity metric between diseases, we compute the Pearson correlation between disease-specific genetic variation profiles. Pearson correlation, ranging from −1 to +1, reflects the strength of a linear relationship between two variables. The correlation coefficient is positive between two diseases if the ***GVS*** for both diseases tend to be simultaneously greater than, or simultaneously less than, their respective means. The correlation coefficient is negative if ***GVS*** for a pair of diseases tend to lie on opposite sides of their respective means. Although other approaches might be considered more robust to outliers, the method we choose to apply relies on the actual ***GVS*** scores as opposed to a ranked ordering of them. Since ***GVS*** scores directly reflect the strength of association between SNPs and disease, computing a similarity metric on the rank ordering of ***GVS*** would result in information loss. Multiple hypothesis testing was not applied to the p-values prior to the calculation of ***GVS*** to keep our methodology simple, since scaling the p-values by a constant amount (such as in Bonferroni correction) would not change our calculated correlation coefficients.

In order to measure significance of the computed correlation coefficients, we re-compute the correlations on randomized data and compute a false discovery rate (FDR) for each actual correlation. Specifically we create the randomized distribution by shuffling SNP labels for each disease and re-compute the pair-wise Pearson correlations between the disease profiles. The randomization is carried out 100 times. The density plot comparison between the actual distribution of correlation coefficients and the ones generated from randomized data are shown in Figure S8.

When computing the false discovery rates, we consider the positive and the negative correlations separately. For each actual correlation score, we count the fraction of top correlations from all randomizations which are at least as extreme as the one we are examining. The false discovery rates based on the randomized distribution are reported in Table S2. We conservatively consider a correlation between two disease profiles to be significant at a false discovery rate of 0.01.

To identify disease classes, hierarchical cluster analysis is applied to the data using the computed Pearson correlation coefficients as a distance metric between disease pairs. Initially, each disease is assigned to its own cluster. The algorithm proceeds iteratively, at each stage joining the two most similar clusters, until there is just a single cluster left. We use the *Pvclust* R package [54] to compute a bootstrap analysis of the clusters. The bootstrap probability of a cluster corresponds to the frequency with which the cluster appears in bootstrap samples of the data. Approximately Unbiased (AU) probability values are computed using bootstrap samples of various sizes and indicate how strongly the cluster is supported by data (AU>95%).

## Supporting Information

Figure S1 Distribution of Commonly Measured SNPs. The distribution of the genomic locations of 573 SNPs that are commonly measured across all the datasets we examine for our analysis.
(0.17 MB PDF)

Figure S2 Genetic Variation Scores for RA (NARAC) and ATD Datasets. Genetic Variation Scores (GVS) for SNPs that are significantly associated with both datasets (p<0.05) are shown in black. The non-significant GVS are shown in gray. The best fit linear regression model of the data is shown in red.
(0.56 MB PDF)

Figure S3 Genetic Variation Scores for RA (NARAC) and MS (WTCCC) Datasets. Genetic Variation Scores (GVS) for SNPs that are significantly associated with both datasets (p<0.05) are shown in black. The non-significant GVS are shown in gray. The best fit linear regression model of the data is shown in red.
(0.64 MB PDF)

Figure S4 Genetic Variation Scores for MS (IMSGC) and AS Datasets. Genetic Variation Scores (GVS) for SNPs that are significantly associated with both datasets (p<0.05) are shown in black. The non-significant GVS are shown in gray. The best fit linear regression model of the data is shown in red.
(0.56 MB PDF)

Figure S5 Genetic Variation Scores for WTCCC and IMSGC MS Datasets. Genetic Variation Scores (GVS) for SNPs that are significantly associated with both datasets (p<0.05) are shown in black. The non-significant GVS are shown in gray. The best fit linear regression model of the data is shown in red.
(0.68 MB PDF)

Figure S6 Genetic Variation Scores for WTCCC and NARAC RA Datasets. Genetic Variation Scores (GVS) for SNPs that are significantly associated with both datasets (p<0.05) are shown in black. The non-significant GVS are shown in gray. The best fit linear model of the data is shown in red.
(0.70 MB PDF)

Figure S7 Volcano Plot (log-odds vs. log p-values) for RA (WTCCC). This plot shows the typical relationship between log-odds ratios and log p-values for an association study. There is no clear relationship between the two measures, meaning that a SNP with a good log-odds ratio, might have a non-significant p-value and a SNP with a significant p-value might have a small odds-ratio.
(0.18 MB PDF)

Figure S8 Randomization based on Genetic Variation. Distribution of correlation scores between pairs of diseases. The distribution based on actual data is shown in red. The distribution of correlations based on randomized data is shown in blue. These are used to compute the false discovery rate for individual pair-wise disease correlations which are presented in Table S2.
(0.16 MB PDF)

Table S1 Pair-wise disease correlations based on Disease Genetic Variation profiles. Values shown in red indicate FDR less than or equal to 0.01.
(0.05 MB DOC)

Table S2 False discovery rates (FDR) based on randomized data. Values shown in red indicate FDR less than or equal to 0.01.
(0.04 MB DOC)
