# Supplementary figures and images for: Autoimmune Disease Classification by Inverse Association with SNP Alleles
Source: PLoS Genet. 2009 Dec 24;5(12):e1000792. doi: 10.1371/journal.pgen.1000792 (PMC2791168; doi:10.1371/journal.pgen.1000792)

# Distribution of Commonly Measured SNPs

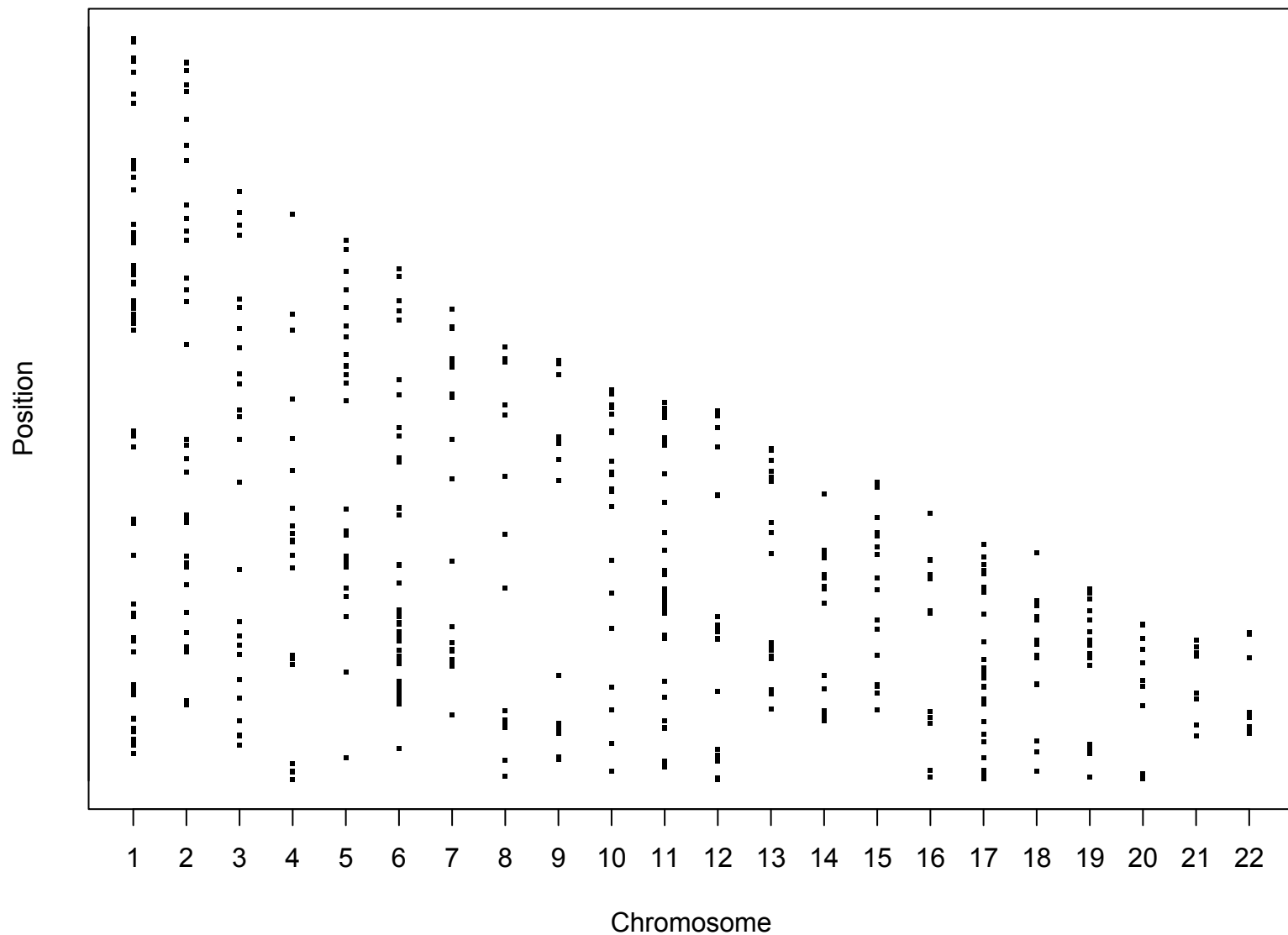

Supplement: Figure S1 — Distribution of Commonly Measured SNPs. The distribution of the genomic locations of 573 SNPs that are commonly measured across all the datasets we examine for our analysis. (0.17 MB PDF) [file pgen.1000792.s001.pdf]

N = 573

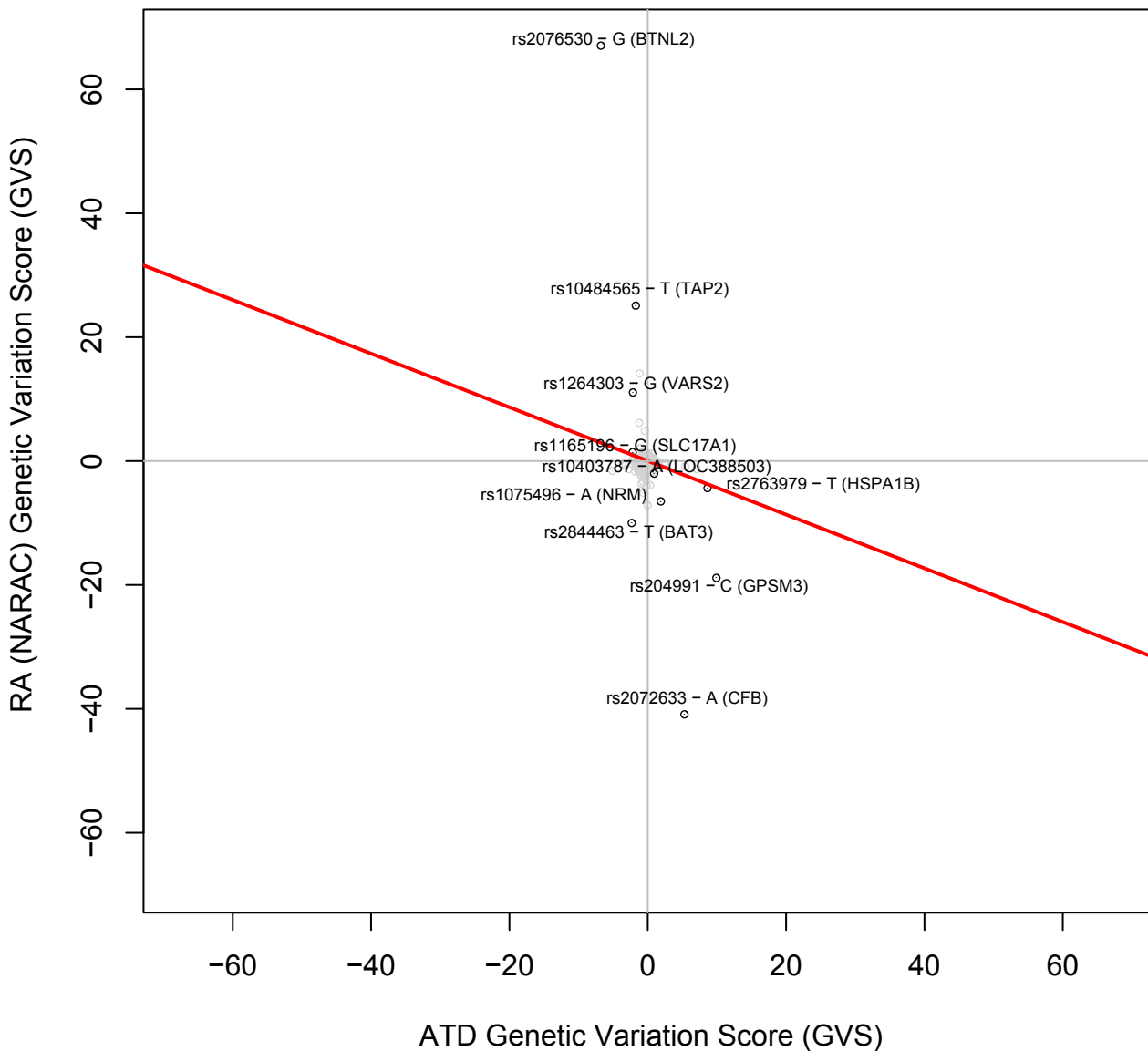

Supplement: Figure S2 — Genetic Variation Scores for RA (NARAC) and ATD Datasets. Genetic Variation Scores (GVS) for SNPs that are significantly associated with both datasets (p<0.05) are shown in black. The non-significant GVS are shown in gray. The best fit linear regression model of the data is shown in red. (0.56 MB PDF) [file pgen.1000792.s002.pdf]

N = 573

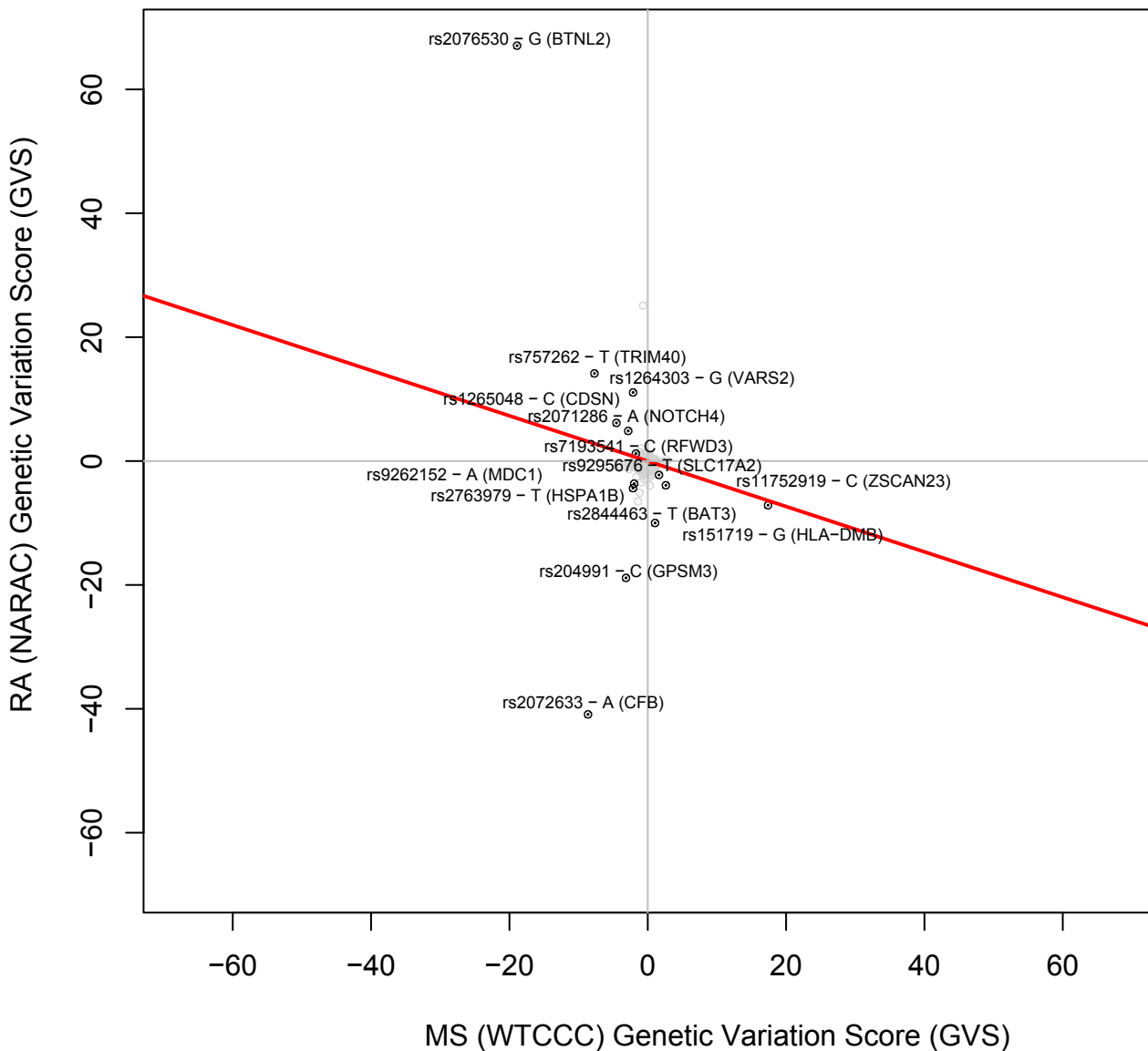

Supplement: Figure S3 — Genetic Variation Scores for RA (NARAC) and MS (WTCCC) Datasets. Genetic Variation Scores (GVS) for SNPs that are significantly associated with both datasets (p<0.05) are shown in black. The non-significant GVS are shown in gray. The best fit linear regression model of the data is shown in red. (0.64 MB PDF) [file pgen.1000792.s003.pdf]

N = 573

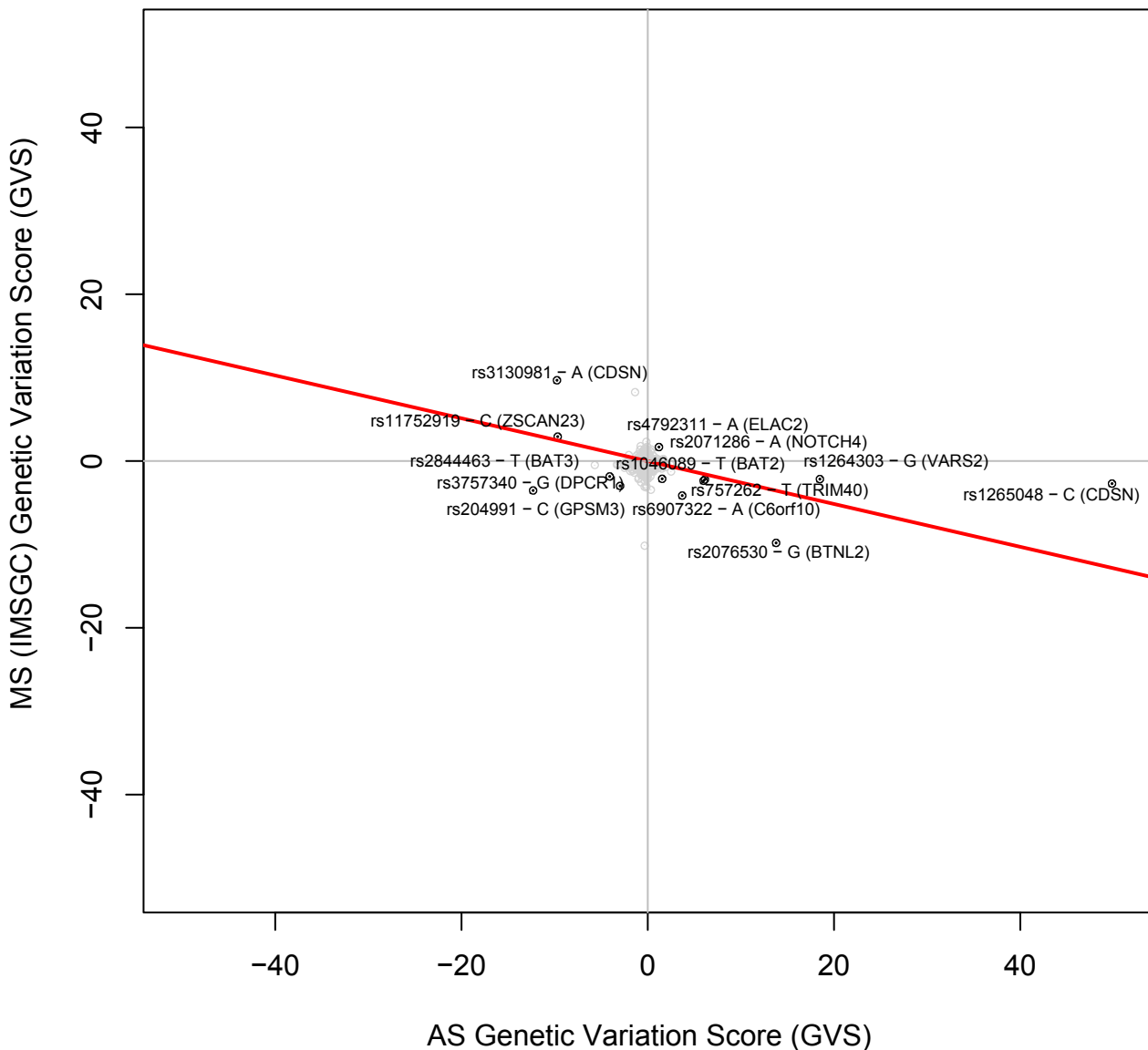

Supplement: Figure S4 — Genetic Variation Scores for MS (IMSGC) and AS Datasets. Genetic Variation Scores (GVS) for SNPs that are significantly associated with both datasets (p<0.05) are shown in black. The non-significant GVS are shown in gray. The best fit linear regression model of the data is shown in red. (0.56 MB PDF) [file pgen.1000792.s004.pdf]

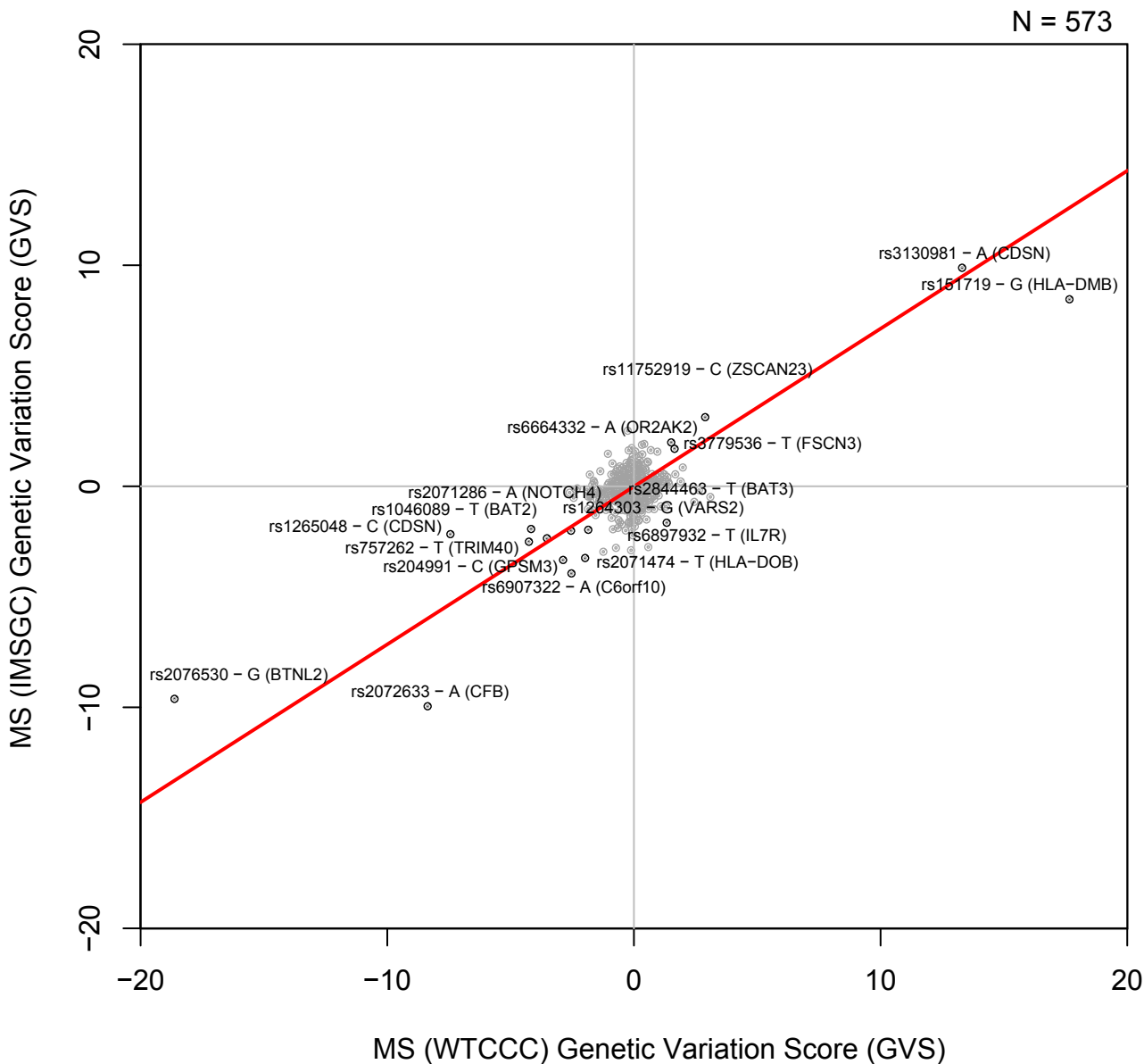

Supplement: Figure S5 — Genetic Variation Scores for WTCCC and IMSGC MS Datasets. Genetic Variation Scores (GVS) for SNPs that are significantly associated with both datasets (p<0.05) are shown in black. The non-significant GVS are shown in gray. The best fit linear regression model of the data is shown in red. (0.68 MB PDF) [file pgen.1000792.s005.pdf]

N = 573

RA (NARAC) Genetic Variation Score (GVS)

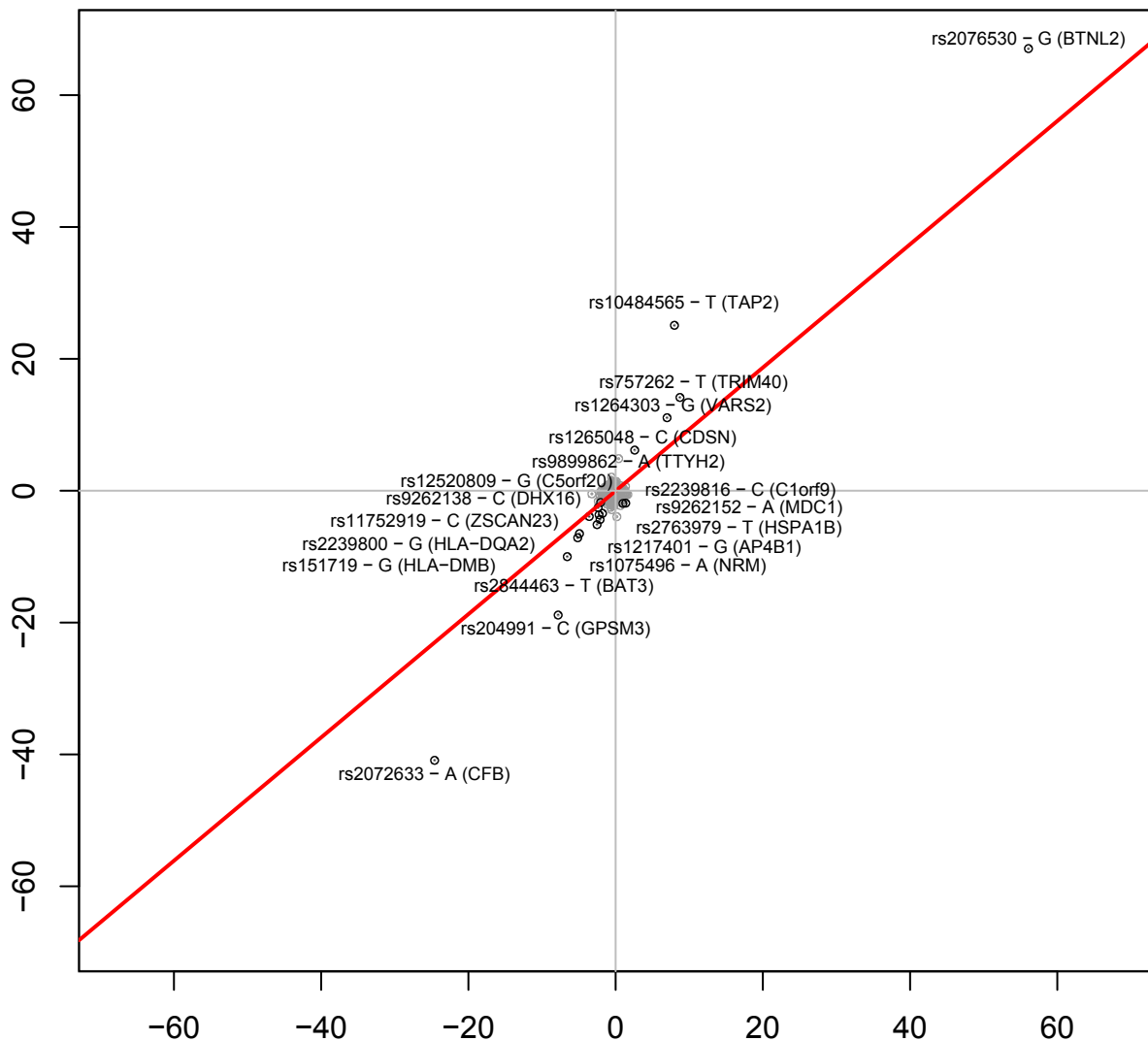

RA (WTCCC) Genetic Variation Score (GVS)

Supplement: Figure S6 — Genetic Variation Scores for WTCCC and NARAC RA Datasets. Genetic Variation Scores (GVS) for SNPs that are significantly associated with both datasets (p<0.05) are shown in black. The non-significant GVS are shown in gray. The best fit linear model of the data is shown in red. (0.70 MB PDF) [file pgen.1000792.s006.pdf]

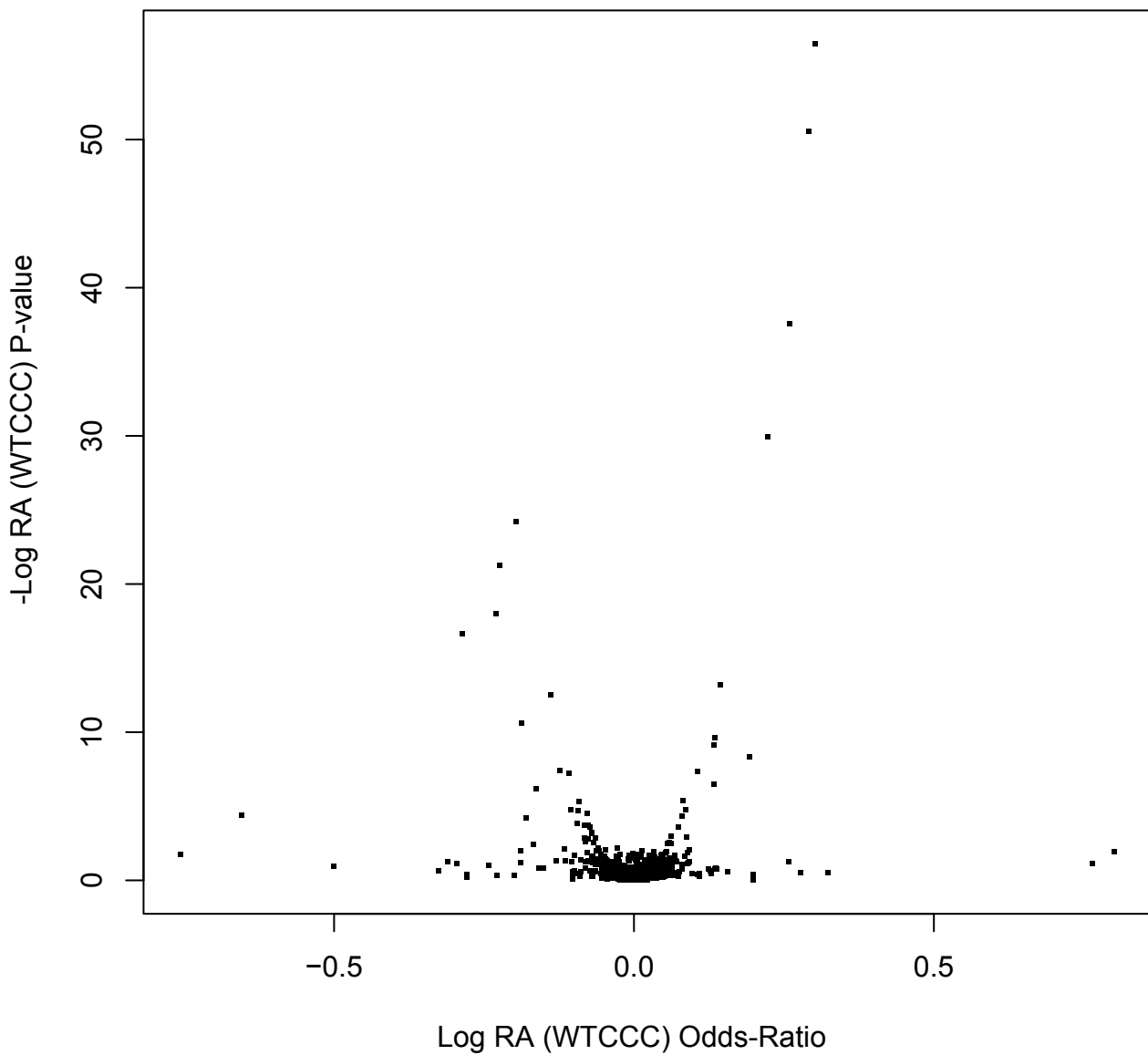

Supplement: Figure S7 — Volcano Plot (log-odds vs. log p-values) for RA (WTCCC). This plot shows the typical relationship between log-odds ratios and log p-values for an association study. There is no clear relationship between the two measures, meaning that a SNP with a good log-odds ratio, might have a non-significant p-value and a SNP with a significant p-value might have a small odds-ratio. (0.18 MB PDF) [file pgen.1000792.s007.pdf]

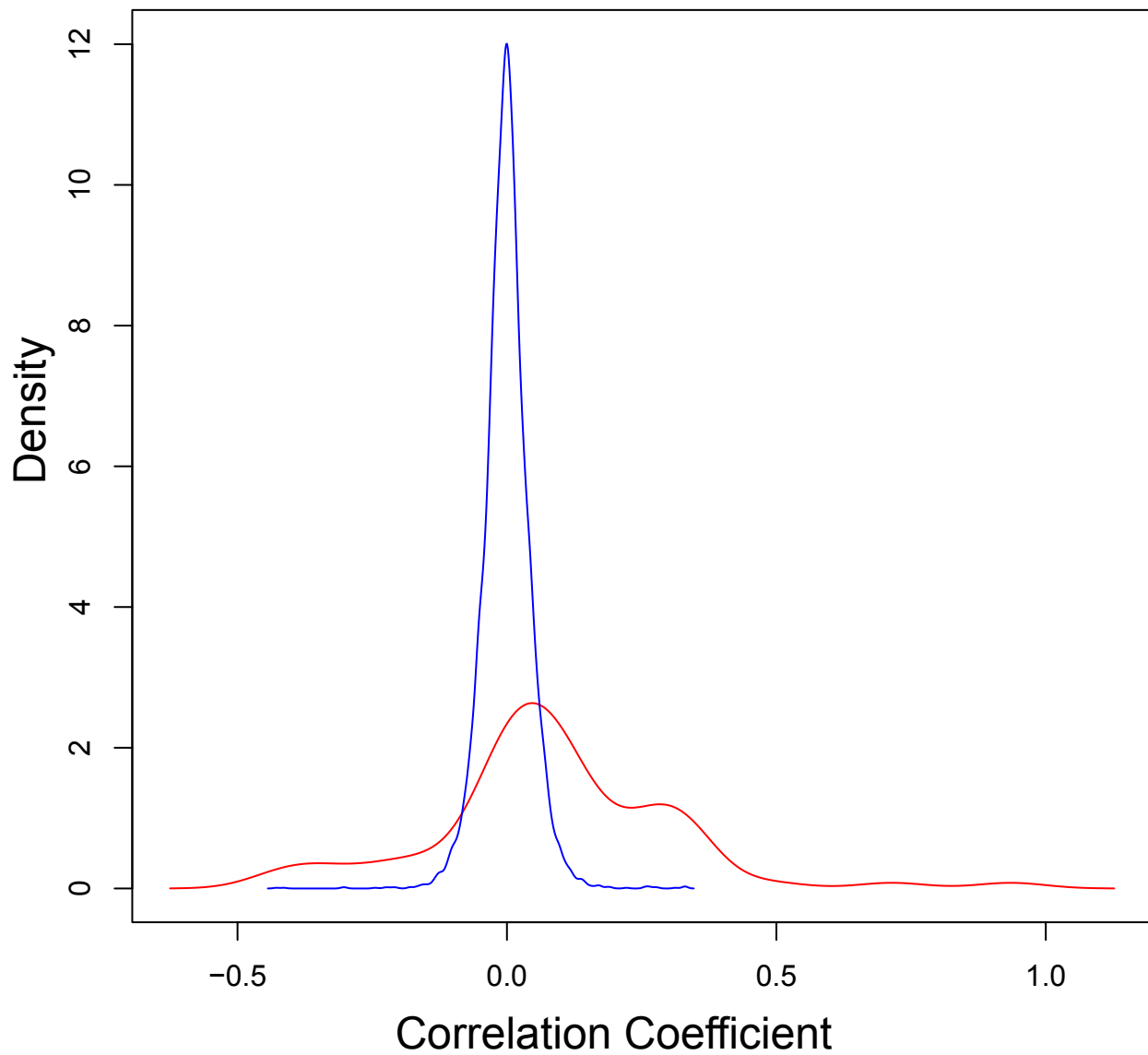

Supplement: Figure S8 — Randomization based on Genetic Variation. Distribution of correlation scores between pairs of diseases. The distribution based on actual data is shown in red. The distribution of correlations based on randomized data is shown in blue. These are used to compute the false discovery rate for individual pair-wise disease correlations which are presented in Table S2. (0.16 MB PDF) [file pgen.1000792.s008.pdf]
