# Supplementary material for: Autoimmune Disease Classification by Inverse Association with SNP Alleles
Source: PLoS Genet. 2009 Dec 24;5(12):e1000792. doi: 10.1371/journal.pgen.1000792 (PMC2791168; doi:10.1371/journal.pgen.1000792)
Supplement: Table S1 — Pair-wise disease correlations based on Disease Genetic Variation profiles. Values shown in red indicate FDR less than or equal to 0.01. (0.05 MB DOC) [file pgen.1000792.s009.doc]

Supplementary Table 1:

| **Pair-wise Disease Correlations Based on Disease Genetic Variation Profiles** | | | | | | | | | | | | | |
| --- | --- | --- | --- | --- | --- | --- | --- | --- | --- | --- | --- | --- | --- |
|  | RA | HT | CD | CAD | T1D | T2D | BD | MS | AS | ATD | BC | NARAC | |
| **HT** | 0.0983 |  |  |  |  |  |  |  |  |  |  | |  |
| **CD** | 0.0781 | 0.3086 |  |  |  |  |  |  |  |  |  | |  |
| **CAD** | 0.1266 | 0.3195 | 0.3327 |  |  |  |  |  |  |  |  | |  |
| **T1D** | 0.1344 | -0.003 | -0.011 | 0.04683 |  |  |  |  |  |  |  | |  |
| **T2D** | 0.1453 | 0.2537 | 0.3088 | 0.34496 | -0.0748 |  |  |  |  |  |  | |  |
| **BD** | 0.1289 | 0.3 | 0.2594 | 0.33036 | -0.0335 | 0.24688 |  |  |  |  |  | |  |
| **MS** | -0.42 | 0.0088 | 0.0674 | 0.00187 | -0.2291 | 0.04445 | -0.0043 |  |  |  |  | |  |
| **AS** | 0.3397 | 0.0823 | 0.0016 | 0.10599 | 0.0661 | 0.09942 | 0.1123 | -0.3222 |  |  |  | |  |
| **ATD** | -0.353 | 0.1205 | 0.124 | 0.14751 | 0.4908 | 0.01695 | 0.0688 | 0.0427 | -0.171 |  |  | |  |
| **BC** | 0.077 | 0.271 | 0.3206 | 0.22879 | 0.0124 | 0.20568 | 0.2284 | 0.0524 | -0.017 | 0.2764 |  | |  |
| **NARAC** | 0.935 | 0.0472 | 0.0265 | 0.05492 | 0.1223 | 0.11168 | 0.0523 | -0.367 | 0.3573 | -0.433 | -0.008 | |  |
| **IMSGC** | -0.204 | -0.039 | 0.008 | -0.05779 | -0.1368 | -0.0277 | 0.0075 | 0.7174 | -0.256 | -0.0059 | 0.0331 | | -0.141 |

Values shown in red indicate FDR less than or equal to 0.01.
