# Supplementary material for: Autoimmune Disease Classification by Inverse Association with SNP Alleles
Source: PLoS Genet. 2009 Dec 24;5(12):e1000792. doi: 10.1371/journal.pgen.1000792 (PMC2791168; doi:10.1371/journal.pgen.1000792)
Supplement: Table S2 — False discovery rates (FDR) based on randomized data. Values shown in red indicate FDR less than or equal to 0.01. (0.04 MB DOC) [file pgen.1000792.s010.doc]

**Supplementary Table 2:**

| **False Discovery Rates (FDR) based on Randomized Data** | | | | | | | | | | | | |
| --- | --- | --- | --- | --- | --- | --- | --- | --- | --- | --- | --- | --- |
|  | RA | HT | CD | CAD | T1D | T2D | BD | MS | AS | ATD | BC | NARAC |
| **HT** | 0.62 |  |  |  |  |  |  |  |  |  |  |  |
| **CD** | 0.95 | 0.04 |  |  |  |  |  |  |  |  |  |  |
| **CAD** | 0.33 | 0.03 | 0.02 |  |  |  |  |  |  |  |  |  |
| **T1D** | 0.27 | 1 | 1 | 1 |  |  |  |  |  |  |  |  |
| **T2D** | 0.25 | 0.06 | 0.04 | 0.02 | 0.97 |  |  |  |  |  |  |  |
| **BD** | 0.33 | 0.05 | 0.06 | 0.02 | 1 | 0.06 |  |  |  |  |  |  |
| **MS** | 0 | 1 | 0.99 | 1 | 0.01 | 1 | 1 |  |  |  |  |  |
| **AS** | 0.02 | 0.91 | 1 | 0.51 | 0.99 | 0.6 | 0.43 | 0 |  |  |  |  |
| **ATD** | 0 | 0.36 | 0.33 | 0.24 | 0.01 | 1 | 0.99 | 1 | 0.05 |  |  |  |
| **BC** | 0.95 | 0.06 | 0.03 | 0.07 | 1 | 0.08 | 0.07 | 1 | 1 | 0.06 |  |  |
| **NARAC** | 0 | 1 | 1 | 1 | 0.34 | 0.43 | 1 | 0 | 0.02 | 0 | 1 |  |
| **IMSGC** | 0.04 | 1 | 1 | 1 | 0.14 | 1 | 1 | 0 | 0.01 | 1 | 1 | 0.11 |

Values shown in red indicate FDR less than or equal to 0.01.
